# *Moringa oleifera* and Propolis in Cattle Nutrition: Characterization of Metabolic Activities in the Rumen In Vitro

**DOI:** 10.3390/metabo12121237

**Published:** 2022-12-09

**Authors:** Mubarik Mahmood, Hasan Ersin Samli, Arife Sener-Aydemir, Suchitra Sharma, Qendrim Zebeli, Ratchaneewan Khiaosa-ard

**Affiliations:** 1 Animal Nutrition Section, Department of Animal Sciences, University of Veterinary and Animal Sciences, Lahore, Sub Campus Jhang, 12 km Chiniot Road, Jhang 35200, Pakistan; 2 Department of Animal Science, Faculty of Agriculture, Tekirdag Namik Kemal University, Degirmenalti Campus, Tekirdag 59030, Turkey; 3 Institute of Animal Nutrition and Functional Plant Compounds, Department for Farm Animals and Veterinary Public Health, University of Veterinary Medicine Vienna, Veterinaerplatz 1, 1210 Vienna, Austria

**Keywords:** moringa seed cake, moringa leaf, propolis, functional compounds, ruminal fermentation metabolite, methane

## Abstract

*Moringa oleifera* by-products such as seed cake and leaves are protein-rich ingredients, while raw propolis has the potential to influence ruminal protein metabolism. These substances are also known to be sources of functional compounds. With these properties, they could modulate ruminal fermentation activities. Using the rumen simulation technique, we investigated ruminal fermentation and the antioxidant properties of four dietary treatments. These included a control diet (CON) without supplementation; the CON diet top-dressed on a dry matter (DM) basis, either with moringa seed cake (MSC, containing 49% crude protein (CP)), moringa leaf powder (ML, containing 28% CP), or raw propolis (PRO, 3% CP). MSC, ML, and PRO accounted for 3.8, 7.4, and 0.1% of the total diet DM, respectively. Both ML and MSC resulted in 14 and 27% more ammonia concentration, respectively than CON and PRO (*p* < 0.05). MSC increased the propionate percentage at the expense of acetate (*p* < 0.05). Both ML and MSC decreased methane percentages by 7 and 10%, respectively, compared to CON (*p* < 0.05). The antioxidant capacity of the moringa seed cake, moringa leaf powder, and raw propolis were 1.14, 0.56, and 8.56 mg Trolox/g DM, respectively. However, such differences were not evident in the fermentation fluid. In conclusion, the supplementation of moringa seed cake desirably modulates rumen microbial activities related to protein and carbohydrate metabolism.

## 1. Introduction

With the help of microbial fermentation taking place in their reticulorumen, ruminants can remarkably utilize low-quality feed ingredients that are not suitable for human consumption. The feedstuff is degraded to various metabolites in the rumen, which fulfill the majority of the nutrient demand of ruminant animals [1]. Suboptimal protein feeding is prevalent in tropical and subtropical areas of the globe, where most of the livestock follows semi-intensive or non-intensive feeding practices [2]. Low dietary protein levels not only limit the availability of the microbial protein to the host animal but also negatively impact their activities to ferment carbohydrates, and thus, suppress the production of total short-chain fatty acids (SCFA) [3] and consequently the productivity of the host animal. Including protein-rich alternatives as well as increased efficiency of nitrogen metabolism in the rumen are options to fill the animal requirement gap.

The *Moringa oleifera* plant is native to tropical and subtropical regions and is a storehouse for a variety of nutrients in sufficient quantities [4]. The best-known benefit is its high crude protein (CP) contents, which are comparable to those of other common protein fodders such as soybeans and alfalfa [5]. A rising trend in the cultivation of moringa plants worldwide during recent years [6] has generated more foliage and seed portions. Moringa seeds are used to extract moringa oil, leaving behind the seed cake that is richer in protein than those of the leaves, seeds, and pods of the parent plant [7,8]. Besides being nutritious, moringa products are also known for harboring a variety of functional compounds, including myricetin, quercetin, moringyne, vanillin, rutins, tannins, gallic acid, and kaempferol [9]. Many compounds present in moringa hold anti-inflammatory, antibiotic, and, importantly, antioxidant properties [10]. However, the composition and quantity of the functional compounds vary greatly among leaf and seed portions of the plant [9]. For instance, moringa leaves contain slightly higher tannins (1.19 mg/g) in comparison to moringa seeds (0.89 mg/g) [11,12]. The role of moringa in cattle nutrition is mainly seen as protein supplements; notably, studies have indicated that moringa by-products may also have functional potential, for instance, in decreasing methanogenesis [13,14], but these are short-term studies. Another functional substance of interest when feeding low-protein diets is raw propolis, which is a non-proteinaceous substance prepared by honeybees from materials of plant origin. Its ammonia (NH_3_)-reducing effect in vitro was reported previously [15]. They suspected that the effect was possibly associated with the reduced deamination of amino acids. This suggests that propolis supplementation might be beneficial in reducing N loss via ammonia production. In addition, propolis has been shown to have antioxidant, antibacterial, and anti-inflammatory effects [16,17,18]. However, due to limited data on moringa and even more lacking raw propolis, their roles as functional feeds for cattle cannot be generalized. In the present study, we aimed to characterize the metabolic activities of ruminal microbiota in response to the supplementation of different moringa by-products and raw propolis. We hypothesized that incorporating these by-products into low-protein diets would improve the ruminal protein metabolism and modulate carbohydrate fermentation, which may suggest their functional effects in cattle nutrition. Using the in vitro rumen simulation technique (RUSITEC), we evaluated the effects of dried moringa leaf powder, moringa seed cake, and raw propolis when supplemented with a low protein diet on ruminal fermentation characteristics and antioxidant capacity.

## 2. Materials and Methods

### 2.1. Experimental Design and Treatments

The trial was performed using two RUSITEC systems, each consisting of six fermenters. Each fermenter had an effective volume capacity of 800 mL. The RUSITEC systems simulated the ruminal conditions by maintaining an anaerobic condition, a temperature of 39.5 °C, and a continuous infusion of a salivary buffer throughout the trial. The trial consisted of two experimental runs in a changeover design [19]. Each run lasted 10 days, with five days of adaptation and system equilibration and the last five days of the sampling period. In each experimental run, we tested four dietary treatments in triplicates: the control (CON), moringa seed cake (MSC), moringa leaves (ML), and propolis (PRO). The CON diet contained hay and a grain mix (50:50 on a dry matter (DM) basis) and was without any supplementation. ML and MSC diets were the CON diet top-dressed with dried moringa leaf powder (containing 28% CP on a DM basis) and moringa seed cake (containing 49% CP on a DM basis), respectively, resulting in the inclusion level of 7.4 and 3.8% of the total diet DM, respectively (Table 1).

The chosen supplementation levels were aimed at providing similar dietary CP content boosting 1.2–1.4% units from that of CON to reach the target CP content of around 11–12%, which was shown to be adequate for milk production under 20 kg/day [20]. These scenarios are typical in tropical and subtropical regions utilizing extensive farming. The PRO diet was a CON diet top-dressed with raw propolis at the rate of 0.1% of the diet DM. The moringa by-products were products of Nicaragua origin and were provided by a private supplier (see Acknowledgments). Propolis, which was a brown type, was obtained from a local honeybee-keeping supplier in Tekirdağ, Turkey. To our knowledge, there was no reference dosage of raw propolis in the cattle reported in the literature. Therefore, the test dosage used in our study was adapted from Santos et al. [21], who used a dried propolis extract plus excipient. The ingredients and chemical composition of all four dietary treatments are shown in Table 1. Before use, the hay and concentrate were ground with a Wiley mill (Pulverisette 25/19; Fritsch GmbH, Idar-Oberstein, Germany) to pass through a 6 mm sieve.

### 2.2. RUSITEC Procedure

On the first day of each experimental run, all fermenters were inoculated with rumen fluid, and solid digesta obtained from 2 ruminally cannulated non-pregnant dry cows (one Holstein cow and one Brown Swiss cow) kept at the ruminant clinic of the University of Veterinary Medicine (Vetmeduni), Vienna, Austria. The cows were fed hay ad libitum with a daily allowance of 0.5 kg of commercial concentrates (KuhKorn PLUS Energie, Garant-Tiernahrung GmbH, Pölchlarn, Austria). They were maintained according to the Austrian guidelines for animal welfare [22]. The inoculation protocol was followed as previously described by Mahmood et al. [19]. Specifically, inoculum from both donor cows was prepared by straining through 4 layers of medical gauze, which were pooled into one batch before inoculation. Subsequently, a total of 600 mL of pooled rumen fluid was transferred into each fermenter already containing 100 mL of McDougall’s buffer (NaHCO_3_, Na_2_HPO_4_·2H_2_O, NaCl, KCl, CaCl_2_·2H_2_O, and MgCl_2_·6H_2_O at 95.1, 23.6, 8.04, 7.64, 0.37, and 0.63 mmol/L, respectively). Equal amounts of the solid digesta from both donor cows were taken, pooled, and used to inoculate the fermenter. The pooled solid digesta and the respective diet containing 12 g DM were packed into separate nylon bags and placed into the respective fermenter. The dimensions of each nylon bag were 120 × 70 mm with a pore size of 70 µm (Linker Industrie-Technik GmbH, Kassel, Germany). Each fermenter was connected to a gas-tight bag for the collection of fermentation gases (TecoBag 8 L, Tesseraux-Spezialverpackungen GmbH, Bürstadt, Germany), and a glass bottle for collecting the outflow was constantly kept cool at 1 °C to prevent further fermentation. After inoculation and the placement of feed bags, each fermenter was closed and flushed with a stream of nitrogen gas for 3 min to establish an anaerobic condition. Throughout the trial, the McDougall’s buffer was continuously infused into each fermenter using a multi-channel peristaltic pump (model ISM932, Ismatec, Idex Health & Science GmbH, Wertheim, Germany) at a flow rate of 375 mL/day. On the next day, the nylon bag with solid rumen digesta was replaced by a new nylon bag containing the respective diet. Before removal, the bag was rinsed and squeezed with 40 mL of a pre-warmed McDougall’s buffer. Before the opening of the fermenter, nitrogen gas was flushed for 30 s to collect all the entrapped fermentation gases into the gas bag, followed by gas bag exchange. The associated effluent bottle was emptied and reconnected. Finally, the fermenter was again made air-tight, and nitrogen gas was flushed for 3 min to re-establish anaerobic conditions. The procedure was performed daily, and each feed bag, which was incubated for 48 h, was daily replaced with a new feed bag of the same treatment.

### 2.3. Sampling, Daily Measurements and Laboratory Analyses

During sampling days, the incubation fluid was collected daily from each fermenter for measurements and analyses. One portion of the aliquot was immediately measured for pH, and the redox potential using a pH meter (Seven Multi TM; Mettler-Toledo GmbH, Schwerzenbach, Switzerland) furnished with separate electrodes: InLab Expert Pro-ISM for pH and Pt 4805-DPA-SC-S8/120 for redox (Mettler-Toledo GmbH, Vienna, Austria). Additionally, another portion of the aliquot was preserved at −20 °C for the later analysis of SCFA and NH_3_. Feed bags taken on the sampling days were rinsed using a machine wash for 30 min with cold water, a gentle cycle mode, and no spinning. The washed bags were manually squeezed to remove excess water and then stored at −20 °C for later chemical analysis.

For the chemical analysis of the incubated feed samples, the feed bags collected across the last 5 days were freeze-dried, pooled per fermenter, and then ground, passing through a 0.75 mm sieve prior to analysis. The ground material was used for analyzing the chemical composition, including the DM, organic matter (OM), CP, ether extract (EE, i.e., crude fat), neutral detergent fiber (NDF), ash, and non-fiber carbohydrates (NFC) and using previously described protocols [23]. Shortly, DM was determined after oven drying at 103 °C and ash after combustion at 580 °C overnight. EE was analyzed using a soxhlet extractor (Extraction System B-811, Buchi, Flawil, Switzerland) and CP using Kjeldahl’s method. The amylase-treated NDF was determined using Fiber Therm FT 12 (Gerhardt GmbH & Co. KG, Königswinter, Germany). The OM was calculated based on the ash percentage. NFC calculation was estimated as follows: NFC = 100 − (CP + ash + EE + NDF). The same chemical analysis was performed on the original diets. The nutrient degradation (% of supply) was based on the apparent nutrient disappearances and was estimated from the differences between the nutrient concentrations before (original diet) and after incubation (feed residue) relative to the supply amount in the original diet times 100.

The analysis of SCFA concentration and the profile of incubation fluid was performed using gas chromatography (GC) and a GC apparatus (Shimadzu GC 2010-Plus, Shimadzu, Kyoto, Japan) equipped with a flame-ionization detector and a 30 m × 0.53 mm i.d. × 0.53 μm capillary column (Trace TR Wax, Thermo Fisher Scientific, Waltham, MA, USA). The quantification of the identified SCFA was conducted using an internal standard (4-methylvaleric acid, Sigma-Aldrich, St. Louis, MO, USA). Helium was used as a carrier gas and was maintained at a flow rate of 6 mL/min. The injector temperature was set at 170 °C while that of the detector was at 220 °C. The indophenol reaction method [24] was used to determine the daily NH_3_ concentrations of the incubation fluid. Accordingly, the preserved samples were thawed at room temperature prior to centrifugation at 15,115× *g* for 10 min. The supernatant was diluted with deionized water to obtain the concentration range within the standard calibration curve. Sodium hydroxide was used to oxidize the phenol and NH_3_ in the presence of dichloroisocyanuric acid and sodium nitroprusside. The absorbance of the treated samples was measured at 655 nm with a spectrophotometer U3000 (INULA GmbH, Vienna, Austria).

The volume of the fermentation gas was estimated by a water replacement method, as described by Soliva and Hess [25]. The composition of the fermentation gases (CH_4_ and carbon dioxide (CO_2_)) was determined with the help of an infrared detector machine (ATEX Biogas Monitor Check BM 2000, Ansyco, Karlsruhe, Germany). Afterward, the absolute production of CH_4_ and CO_2_ (mL/day) was calculated.

The ferric reducing antioxidant power (FRAP) assay was performed on original materials (moringa leaf powder, moringa seed cake, and raw propolis) and daily samples of the incubation fluid followed the procedure of Benzie and Strain [26] with minor modifications. Shortly, 24 µL each of the blank, standard, and sample were transferred in duplicates into a 96-well plate, followed by the addition of 180 µL of the pre-warmed (37 °C) working reagent. The working reagent consisted of 25 mL of acetic acid buffer, 2.5 mL of TPTZ (2,4,6-tripyridl-s-triazin) solution, and 2.5 mL of FeCl_3_·6H_2_O. The absorbance was measured at 490 nm after 5 min of reaction time using a thermostat spectrophotometer (xMark, Bio-Rad). A calibration curve with an increasing Trolox concentration in the range of 0–9.6 µg/24 µL was used for the quantification to express the results in Trolox equivalents.

### 2.4. Statistical Analysis

Statistical analysis was performed using the MIXED procedure of SAS (version 9.4, SAS Institute Inc., Cary, NC, USA). There were two kinds of data: daily data in the case of fermentation characteristics and fermentation gas formation and one-time data (pooled feed bags) for nutrient degradation. For the daily data, repeated measures of ANOVA were used to compare the fixed effect of the treatments on the fermentation characteristics and fermentation gas formation. The variation among the experimental runs was regarded as a random effect. The measurement day was the repeated measure factor, and compound symmetry was the variance-covariance structure. For the one-time data, one-way ANOVA was used to compare the effect of the treatments on nutrient degradation. The variation between experimental runs was regarded as a random effect. Pairwise comparisons between the treatments were carried out using Tukey’s test. The significance was declared at *p* ≤ 0.05, whereas the tendency of an effect was observed at 0.05 ≤ *p* ≤ 0.1.

## 3. Results

The nutrient disappearances are summarized in Table 2. Overall, no difference among the treatments was detected for the degradation of DM, OM, EE, CP, NDF, and NFC. Only the treatment ML lowered the ash disappearance compared to the other groups (*p* < 0.05).

Table 3 illustrates the ruminal fermentation characteristics as affected by the treatment. While the pH of the incubation fluid was unaffected, both ML and MSC groups lowered their redox potential compared to CON and PRO (*p* < 0.05). There was an increase in the NH_3_ concentration (mmol/mL) with both ML (+14%) and MSC (+27%) in comparison to CON and PRO (*p* < 0.05). Treatment tended to affect the concentration of SCFA (*p* = 0.06).

According to Tukey’s test, MSC resulted in 10.5% higher SCFA concentration compared to PRO (*p* < 0.10), while CON and ML showed intermediate values. MSC showed the strongest shift in the SCFA composition compared to CON (Table 3). Specifically, MSC increased propionate at the expense of the acetate (*p* < 0.05), thereby significantly reducing the acetate to a propionate ratio in comparison to the other treatments. The relative proportions of butyrate and caproate were significantly uplifted exclusively with PRO in comparison to that of CON (*p* < 0.05). The percentage of isobutyrate (*p* = 0.01) and heptanoate (*p* < 0.001) were also affected by the treatment, while valerate and isovalerate were unaffected.

**Figure 1:**
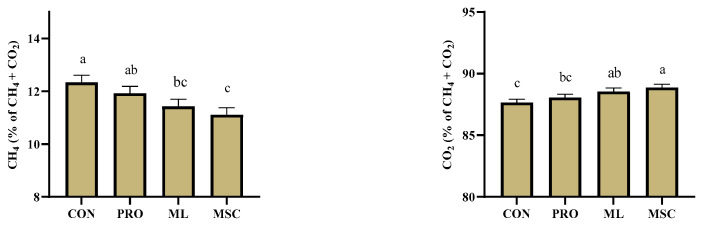
Relative proportion of carbon dioxide (CO_2_) and methane (CH_4_) as affected by PRO, ML, and MSC. Experimental diets included a diet with concentrate at 500 g kg^−1^ of diet dry matter without supplementation (CON) or top-dressed with propolis (PRO), dried moringa leaves (ML), or moringa seed cake (MSC). ^abc^ Least square means sharing no common superscripts differed significantly (*p* ≤ 0.05) according to Tukey’s method.

Treatment did not affect the absolute production (mL/d) of the total fermentation gases or individual CH_4_ and CO_2_ but affected the relative proportion (% of total gas) of both CO_2_ (*p* = 0.02) and CH_4_ (*p* < 0.001) (Table 3, Figure 1). The inclusion of the moringa by-products increased the estimated gross energy intake (*p* < 0.001) but decreased the methane conversion rate (MCR) relative to the gross energy intake (*p* = 0.020).

Accordingly, both ML and MSC resulted in 7% and 10% lower CH_4_ percentages at the expense of CO_2_ compared to CON (*p* < 0.05), respectively, while PRO did not show any difference from CON. As analyzed using the FRAP assay, the antioxidant capacity of the moringa seed cake, moringa leaf powder, and propolis was 1.14, 0.56, and 8.56 mg Trolox/g DM. However, the treatment did not affect the antioxidant capacity of the incubation fluid (Table 3).

## 4. Discussion

Moringa seed cake and moringa leaves are protein-rich ingredients that can be successfully used in ruminant diets. Moringa seed cake is a by-product obtained during the acquisition of oil from moringa seeds. Moringa seed cake and moringa leaves have been used as a substitute for good quality protein sources such as soybean meal [5,28]. The current data indicate that both moringa by-products promote the rumen microbial fermentation of proteins and carbohydrates, albeit the effect of MSC was often more prominent than that of ML. The most apparent effect of MSC and ML was the increased NH_3_ concentrations (+27 and +14% of that of CON, respectively). Since the ML and MSC did not affect the CP degradation, the boosting effect of the NH_3_ concentration was, therefore, associated with the increased substrate (CP) in the diet. Notably, the moringa seed cake and moringa leaves contained similar proportions of rumen degradable protein (58.4% and 66.8% of total CP, respectively) [29], which may explain the similar CP disappearances between ML and MSC observed in the present study. A higher inclusion rate (40% of diet DM) of the moringa seeds led to a greater increase in the ruminal NH_3_ concentration [30] compared to the present findings. Karim et al. [12] documented the positive impact of moringa leaves on the characteristics of protein fermentation. The moringa treatments numerically increased the total SCFA concentration, which was possibly due to the extra dietary nutrients as well as an improvement in the protein and energy balance of the diet, which is important for microbial growth and activity [31]. The MSC diet also profoundly shifted the SCFA proportion to more propionate and less acetate. Aboamer et al. [32] evaluated the effect of moringa seed cake as a substitution for cottonseed meal on nutrient digestibility and milk production in Ossimi ewes. They found that the inclusion level of 2.5% of the diet DM increased gas production in vitro and increased milk lactose concentration in the ewes. Their findings were in line with the propionate-boosting effect observed in the present study. Propionate is a glucogenic precursor that is required for lactose synthesis [33]. On the contrary, the ML diet did not alter the SCFA profile despite the higher inclusion rate of moringa leaf powder compared with moringa seed cake. In agreement, Soliva et al. [34] observed no change in the SCFA production and composition even with the inclusion level as high as 30% of the diet DM. The different findings between MSC and ML hinted that the alteration of SCFA pathways might result from some secondary compounds unique to moringa seed cake, for instance, moringyne and vanillin [9]. Interestingly, ML decreased with the ash disappearance. The ruminal disappearance of minerals depended on the solubility of the minerals [35]. Our data may indicate a lower solubility of the minerals in the moringa leaf powder compared to the seed cake, which is likely due to the higher oxalate contents in moringa leaves than in the seed (1050 vs. 2.9 mg/100 g, respectively) [36].

We revealed that moringa by-products, especially seed cake, could have an added function in mitigating CH_4_. It must be pointed out that the treatment difference in the absolute yield of CH_4_ (mL/d) did not reach statistical significance. However, the absolute yield was also confounded with the top-dressing strategy that increased the amounts of substrates in moringa treatments. When the methane production was standardized by the diet, i.e., MCR (% of gross energy intake) or calculated as a proportion relative to the total gas production, the effect of moringa by-products became evident; therewith, MSC showed the strongest decrease in both variables. The CH_4_-lowering effect observed with MSC could be explained by the increase in propionate, which is a metabolic hydrogen sink in the rumen [37], therefore, reducing the availability of metabolic hydrogen for methanogenesis. In line with our findings, the in sacco study revealed that out of seven different seed cakes from moringa, castor, cotton, palm kernel, radish, soybean, and sunflower, only moringa seed cake expressed a CH_4_ mitigating property [13]. Their inclusion level was 40% of DM, which is about 10 times higher than the inclusion level used in the present study. This hints that low dosages of moringa seed cake can be effective in mitigating CH_4_, which in turn can be attributed to the presence of oil in moringa seed cake, as oils likely reduce methanogenesis [38]. Surplus lipids could be detrimental to ruminal microbes especially those degrading fiber [39]. However, this was not the main explanation for the effect of MSC observed in the present study because the EE levels of all the diets (approx. 2% of diet DM) were still within the range deemed suitable for rumen microbial fermentation [40]. Moringa contains various functional compounds, including myricetin, quercetin, moringyne, vanillin, rutins, tannins, gallic acid, and kaempferol [9], which may contribute to the CH_4_-lowering effect of moringa by-products tested in the present study. Some of these compounds in the extracts of other plants have been shown to mitigate enteric CH_4_ production [41,42,43]. The weaker effect of ML on rumen fermentation variables, despite its higher inclusion level than MSC, might be related to the presence of different functional compounds. The contents and profiles of secondary compounds in moringa leaves vary from those of the moringa seed [9,11,12]. Additionally, we showed that moringa seed cake had twice the antioxidant power, based on the FRAP assay, compared to moringa leaf powder. Some plant secondary compounds, such as tannins, are known to reduce CH_4_ synthesis via multiple routes, some of which do not involve an association with propionate production [44].

Despite the higher antioxidant value than those of moringa products, the addition of raw propolis did not drastically modulate the ruminal fermentation and the gas production parameters in the present experiment. Some researchers explored the effects of the propolis extract [14,21,45,46] or propolis phenols [42] and consistently documented higher butyrate production. Data from earlier studies suggest that the polyphenolic compounds of propolis, for instance, caffeic acid [47], might have played a role because it supports the growth of gut butyrate-producing bacteria [48], which might have replaced the population of other bacteria, such as propolis which also possess microbial inhibition properties [49]. Notably, we observed the highest proportion of butyrate with the raw propolis, but the change did not reach significance. This may be related to the low dosage as well as the form, i.e., raw propolis. To our knowledge, there is no comparative study using raw propolis on ruminal fermentation characteristics. In addition, our test product was from a local supplier, and thus, variation in the product quality and the effect on ruminal fermentation must be considered as well. Still, given the role of butyrate as a promoter of gut epithelial integrity [50], future research may invest in finding the effective (higher) dosages of propolis that could express a benefit on ruminal fermentation and gut health.

## 5. Conclusions

The inclusion of moringa seed cake at 3.8% of the diet DM modulated ruminal fermentation characteristics, leading to greater NH_3_, favoring propionate production, and mitigating CH_4_ without any negative effect on nutrient disappearance and physicochemical parameters in vitro. At a higher inclusion rate (7.4% of diet DM), the moringa leaf powder showed a similar direction, albeit a weaker effect, on NH_3_ and CH_4_ variables. Despite having a stronger antioxidant power compared to the moringa by-products, raw propolis supplemented at 0.1% of the diet DM did not affect the fermentation variables in vitro. Our data suggest that feeding moringa seed cake desirably modulates the rumen microbial metabolic activities of proteins and carbohydrates. Of note, in vitro studies do not account for host-dependent influences. In vivo studies are needed to identify the effective dosages that facilitate the health and production of ruminants.

## Figures and Tables

**Table 1:** Ingredient and chemical composition of control and experimental diets (g/kg DM) *.

Item	CON	PRO	ML	MSC
Ingredients				
Meadow hay	503	502	465	483
Concentrate ^1^	497	497	461	478
Propolis	0.0	1.0	0.0	0.0
Moringa leaf powder	0.0	0.0	74	0.0
Moringa seed cake	0.0	0.0	0.0	38
Chemical composition				
Dry Matter	894	894	897	895
Organic Matter	915	915	914	916
Crude Protein	99	99	112	114
Ash	85	85	86	84
Neutral detergent fiber	475	474	457	464
Ether extract (crude fat)	17	17	20	21
Non-fiber carbohydrates	325	325	324	318

* All fermenters were supplied with the same basal diet containing 50:50 hay and concentrate on a dry matter basis. The respective test ingredient was top-dressed. A diet containing concentrate at 500 g/kg of diet dry matter without supplementation (CON) or top dressed in propolis (PRO), dried moringa leaves (ML) or moringa seed cake (MSC). ^1^ Contained 216 barley; 216 wheat; 517 maize; and 52 vitamin and mineral supplement on dry matter basis (g/kg) (Rindavit TMR 11 ASS-CO  +  ATG; H. Wilhelm Schaumann GmbH & Co KG, Brunn/Gebirge, Austria).

**Table 2:** Ruminal nutrient disappearance (percentage of supply) as affected by dietary treatment *.

Item	CON	PRO	ML	MSC	SEM	*p*-Value
Dry matter	42.9	43.0	41.7	42.8	2.0	0.735
Organic matter	40.5	40.6	39.7	40.3	1.9	0.909
Crude protein	43.8	42.9	41.5	43.4	2.4	0.881
Ash	69.13 ^a^	69.30 ^a^	64.11 ^b^	69.45 ^a^	2.69	0.019
Neutral detergent fiber	20.86	19.93	19.71	20.00	3.29	0.277

SEM: standard error of the mean. * A diet containing concentrate at 500 g/kg of diet dry matter without supplementation (CON) or top dressed in propolis (PRO), dried moringa leaves (ML), or moringa seed cake (MSC). ^ab^ The values within the same row with different superscripts indicate a significant difference (*p* < 0.05) according to Tukey’s test.

**Table 3:** Ruminal fermentation parameters as affected by dietary treatments *.

Item	CON	PRO	ML	MSC	SEM ^1^	*p*-Value
pH	6.81	6.82	6.81	6.8	0.01	0.368
Redox potential (mV)	−197 ^a^	−196 ^a^	−254 ^b^	−263 ^b^	6.00	<0.001
Ammonia (mmol/L)	3.75 ^c^	3.73 ^c^	4.26 ^b^	4.75 ^a^	0.12	<0.001
Total SCFAs ^2^ (mmol/L)	69.6 ^xy^	67.2 ^y^	72.4 ^xy^	74.3 ^x^	5.79	0.064
SCFAs profile (mol/100 mol)						
Acetate	49.5 ^a^	49.8 ^a^	49.7 ^a^	48.1 ^b^	0.40	<0.001
Propionate	22.9 ^b^	22.1 ^b^	22.3 ^b^	25.3 ^a^	0.50	<0.001
Butyrate	7.20 ^b^	7.66 ^a^	7.32 ^ab^	7.05 ^b^	0.19	0.008
Isobutyrate	0.66 ^ab^	0.67 ^a^	0.64 ^b^	0.64 ^b^	0.01	0.018
Valerate	9.0	8.90	9.28	9.34	0.20	0.184
Isovalerate	4.27	4.03	4.08	4.37	0.36	0.103
Caproate	4.34 ^b^	4.78 ^a^	4.64 ^ab^	3.63 ^b^	0.25	<0.001
Heptanoate	2.29 ^ab^	2.36 ^b^	2.53 ^a^	2.01 ^b^	0.13	<0.001
Acetate to propionate	2.20 ^a^	2.29 ^a^	2.25 ^a^	1.94 ^b^	0.05	<0.001
Fermentation gases						
Total fermentation gas (mL/d)	386	427	403	387	38	0.685
Carbon dioxide (mL/d)	312.0	347.0	331.0	318.0	33.00	0.738
Methane (mL/d)	44.0	47.0	42.7	39.4	3.8	0.225
^3^ MCR (% Gross energy intake)	0.84 ^xy^	0.90 ^x^	0.75 ^xy^	0.72 ^y^	0.07	0.020
Gross energy intake (MJ/d)	0.207 ^a^	0.207 ^a^	0.226 ^b^	0.218 ^c^	0.0001	<0.001
Antioxidant capacity (µg Trolox/mL) ^4^	2.58	2.77	2.33	2.35	0.31	0.183

* A diet containing concentrate at 500 g/kg of diet dry matter without supplementation (CON) or top-dressed in propolis (PRO), dried moringa leaves (ML) or moringa seed cake (MSC). ^abc^ Least square means sharing no common superscripts differ significantly (*p* ≤ 0.05) according to Tukey’s method. ^xy^ Least square means sharing no common superscripts tend to differ (0.05 < *p* ≤ 0.10) according to Tukey’s method. ^1^ SEM: standard error of the mean. ^2^ Short chain fatty acids. ^3^ Methane conversion rate was estimated as follows: gross energy (Mcal/kg DM) according to Weiss and Tebbe [27], subsequently, gross energy intake (MJ/d) was quantified, and finally, methane production was adjusted to methane in MJ/100 MJ of gross energy intake or %. ^4^ Using ferric reducing antioxidant power (FRAP) assay

## Data Availability

The data presented in this study are available in the main article.
